# A hierarchical Bayesian model to predict APOE4 genotype and the age of Alzheimer’s disease onset

**DOI:** 10.1371/journal.pone.0200263

**Published:** 2018-07-12

**Authors:** Francis Hane, Carolyn Augusta, Owen Bai

**Affiliations:** 1 Lakehead University, Thunder Bay, Ontario, CANADA; 2 Thunder Bay Regional Health Research Institute, Thunder Bay, Ontario, CANADA; 3 University of Guelph, Guelph, Ontario, CANADA; University of North Carolina at Chapel Hill, UNITED STATES

## Abstract

In this work we use a hierarchical Bayesian paradigm to introduce a theoretical framework to determine an individual’s Apolipoprotein ε4 (APOE4) genotype, which heavily influences both the age of onset and probability of acquiring Alzheimer’s disease (AD). This calculation is based solely on an individual’s family history. This APOE4 genotype estimation is then combined with a number of known factors that influence AD onset to produce a function that estimates the onset of AD as a function of age. We disseminated our Alzheimer’s predictive tool online at http://www.alzheimerspredictor.com.

## Introduction

Alzheimer’s disease (AD) is a devastating age-related neurodegenerative disease. Its devastation lies in its ability to impair the memory and cognitive function of people who have lived to an elderly age by having avoided or conquered many of the other mortal diseases such as cancer, cardio- and cerebro-vascular diseases, and respiratory infections. This cognitive impairment requires the allocation of extensive familial and societal resources to care for the afflicted individual [1]. Many people are interested in obtaining genetic testing to determine their possibility of suffering from AD and a variety of corporations have begun to offer these genetic testing services to fulfil the demand. The results of these tests have not been demonstrated to cause significant emotional harm to subjects [2]. Genetic testing involves the collection of blood or other genetic material to detect the presence of the APP, PSEN1, or PSEN2 genes in the case of early onset AD (EOAD) or the APOE-ε4 (APOE4) allele in the case late onset AD (LOAD) [3]. While these genetic tests are very accurate in determining genotype, these tests suffer cost drawbacks as well as a general failure to predict the age of onset of AD by correcting for other factors such as history of Type 2 Diabetes Mellitus (T2DM) and traumatic brain injury (TBI). A number of factors have been demonstrated to affect the probability of AD and the age of onset. Major factors that influence age of AD onset include T2DM [4,5], TBI [6,7], education level [8], and race [9]. Additionally, previous studies have found observational links between age of onset and a variety of foods, mental activity, and physical exercise [10].

A number of other reports have successfully demonstrated the use of various data to predict the onset of AD. Tierney et al used neuropsychological tests to predict AD with a sensitivity and specificity above 70% for predicting AD within 5 years [11]. Callahan et al used clinical memory test scores and imaging biomarkers to derive a predictive model for AD [12]. Macdonald et al derived a mathematical model of AD based on APOE status [13] based largely on the earlier work by Farrar et al. [14].

In this work, we propose a theoretical framework to predicting AD onset by applying a hierarchical Bayesian model to a subject’s familial history to predict their APOE4 genotype, the major genetic risk factor for LOAD. Firstly, this model considers the familial history of the subject dating back two generations, i.e. parents and grandparents. Following the APOE4 genotype estimate, known hazard ratios are applied to determine a mathematical model of the probability of AD as a function of age correcting for covariates sex, race, diabetic history, traumatic brain injury, physical activity, diet, and education level. Important limitations of this approach are discussed in the discussion section of this report.

Our novel AD prediction model provides a theoretical predictive tool that corrects for various factors not considered in existing genetic tests without the costs associated with genetic tests. Our model is accessible online at http://www.alzheimerpredictor.com for general interest purposes.

## Methods

### Predicting the APOE-ε4 genotype

In order to obtain the most information about the APOE4 genotype, we began by estimating the genotype of each grandparent using Bayes theorem [15] to determine the conditional probability that given a particular age of AD onset, the person has either an APOE4 +/+, +/-, or -/- genotype. The APOE4 allele frequencies within the general population of 86.5% for -/-, 9% for +/- and 4.5% for +/+ [16] formed the prior probabilities for our calculations.

The conditional probability of an individual having a certain APOE genotype based on the age of AD onset, between 45 and 95 years of age, P(Genotype|AGE), was solved using Bayes theorem,

$$P(Genotype|AGE,\;{AGE}_{Pat},{AGE}_{Mat})=\frac{P(AGE|Genotype,\;{AGE}_{Pat},{AGE}_{Mat})∙P(Genotype|{AGE}_{Pat},{AGE}_{Mat})}{P(AGE|{AGE}_{Pat},{AGE}_{Mat})}$$ (1)

Where P(AGE|Genotype) is the probability of having AD at a specific age based on the genotype, P(Genotype) is the prior probability of having a specific APOE4 genotype and P(AGE) = Σ (P(AGE|Genotype) · P(Genotype)).

For example, the conditional probability of a subject having an APOE4 +/+ genotype is given by the equation:

$${P({Genotype}_{++}|AGE)}_{}=\frac{P({AGE|Genotype}_{++})P({Genotype}_{++})}{P(AGE)}$$ (2)

Where P(Genotype_++_) is prior probability of an individual having an APOE4 +/+ genotype which is equal to the frequency of the APOE +/+ genotype in the population (i.e. 4.5%).

Within the population, the mean age of onset for APOE4 +/+ individuals is 68±8.2; +/- 76±8.2, and 84±8.2 for -/- individuals [3,16]. Assuming a Gaussian distribution for the age of AD onset, the probability of a given individual, at age, AGE, with a given genotype, with an mean onset age of MeanOnsetAge ± σ, having the onset of AD symptoms was calculated using a Gaussian distribution where,

$$P({AGE|Genotype}_{g})={\left(\sigma_{g}\sqrt{2\pi }\right)}^{-1}exp\;\left[-\frac{{\left(AGE-{MeanOnsetAge}_{g}\right)}^{2}}{2{\sigma_{g}}^{2}}\right]$$ (3)

Where σ_g_ is the standard deviation of the age of onset, AGE is the age of onset, MeanOnsetAge_g_ is the mean age of AD onset. The subscript, *g*, is used to indicate generality of the genotype. To calculate the probability for a given genotype, the values for the genotype being calculated are used. For example, to calculate the probability for the APOE4 +/+ genotype, P(AGE|Genotype_++_), σ_++_ and MeanOnsetAge_++_ are used.

P(AGE) is given by the equation:

$$P(AGE)={PopFreq}_{++}∙P({AGE|Genotype}_{++})+{PopFreq}_{+-}∙P({AGE|Genotype}_{+-})+\;{PopFreq}_{--}∙P({AGE|Genotype}_{--})$$ (4)

Where PopFreq is the population frequency of the occurrence of an allele. These equations are used to calculate the conditional probability of an individual having a given APOE4 genotype as a function of age of onset of AD symptoms.

$${P(Genotype|AGE)}_{g}=\frac{P({AGE|Genotype}_{g})\;∙{PopFreq}_{g}}{P(AGE)}.$$ (5)

We used a hierarchical Bayesian methodology to estimate the posterior probability of each genotype for each subsequent generation. The probability of each genotype for progeny was calculated by multiplying the probabilities of the genotypes of the parents by factors obtained from Mendelian statistics (¼, ½, ¼ for +/- +/- parents) [17,18]. The sum of probabilities for each possible genotype was calculated and was used as the prior probabilities for the next generation.

For example, the prior probability of a subject who developed AD at a specific age having an APOE4 +/+ genotype, regardless of whether or not the parents had AD, is given by:

$$\begin{array}{l} P\left(Genotype_{++}|AGE_{Pat},AGE_{Mat}\right)=\\ \begin{array}{ll} & P\left(Genotype_{Pat++}|AGE_{Pat}\right)\cdot \;P\left(Genotype_{Mat++}|AGE_{Mat}\right)\\ & +\frac{1}{2}\left[P(Genotype_{Pat++}|AGE_{Pat})\cdot \;P(Genotype_{Mat+-}|AGE_{Mat})\right]\\ & +\frac{1}{2}\left[P(Genotype_{Pat+-}|AGE_{Pat})\cdot \;P(Genotype_{Mat++}|AGE_{Mat})\right]\\ & +\frac{1}{4}\left[P(Genotype_{Pat+-}|AGE_{Pat})\cdot \;P(Genotype_{Mat+-}|AGE_{Mat})\right] \end{array} \end{array}$$ (6)

Where the subscripts “*Pat*” and “*Mat*” denote the previous generations’ posterior probabilities that the given allele was inherited from the paternal and maternal side respectively.

Although we calculated the probability of the subject having any of the rare autosomal dominant genotypes (APP, PSEN1, PSEN2), the very small genetic frequency of these genes resulted in a negligible posterior probability at most relevant ages and therefore the consideration of autosomal dominant genetics was omitted from further consideration for our model.

A baseline hazard function was calculated by fitting the cumulative risk of AD to a Gompertz function which is a function known to fit well to AD onset probability [13]. The Gompertz function has a general form of,

$$P(AGE)=A\;\text{exp}\;\left(-exp[k(AGE-x_{b})]\right)$$ (7)

Applying the probabilities of each APOE genotype to the Gompertz function, the function is expanded to:

$$P_{AD}(AGE)=\left(\sum A∙\;P\right)\text{exp}\;\left(-\;\text{exp}\;\left(\left(\sum -k\;∙\;P\;)[AGE-(\sum x\;∙\;P\;)\right]\right)\right)$$ (8)

Where Σ A · P = A_+/+_ · P_+/+_ + A_+/-_ · P_+/-_ + A_-/-_ · P-/-. Σ k · P and Σ x · P follow a similar pattern. P_+/+_, P_+/-_, P_-/-_ is the probability of the respective genotype based on the Bayesian analysis (e.g. P_+/+_ = P(Genotype_++_ | Age)). Coefficients A, k, x are determined by fitting the cumulative AD symptom probability function to a Gompertz function (Table 1).

**Table 1:** 

	APOE ε4 +/+	APOE ε4 +/-	APOE ε4 -/-
**A**	0.9377	0.4962	0.225
**k**	0.137	0.1295	0.116
**x**	64.09	72.294	80.92

Curve parameters for the baseline Gompertz function for different APOE alleles. Notice that the values of A correlate with the risk of AD at any age, and x the mean age of AD onset.

### Gompertz probability function

Following the determination of the probability of the subject having each of the possible APOE4 alleles and fitting this data to a Gompertz function, we applied previously determined hazard ratios to modify Gompertz age of AD onset function. Since hazard ratios were acquired from different sources, we had to adjust the hazard ratios to obtain a “mean” hazard ratio of the population of 1. The mean hazard ratio for a given factor were determined by the following equation:

$${HR}_{adj}=\frac{HR}{\sum \left(HR\;∙PopFreq\right)}$$ (9)

Where HR_adj_ is the adjusted hazard ratio, HR is the hazard ratio, and PopFreq is the percentage of the population with the given phenotype.

The Gompertz function, adjusted for estimated APOE genotype, was then multiplied by a factor equal to the sum of the hazard ratios to obtain a factored age of AD onset function to yield a final factored risk function,

$$P_{AD}{\left(AGE\right)}_{factored}=P_{AD}(AGE)\;∙\;\left[\sum ({HR}_{adj}-1)+1\right]$$ (10)

Justification and limitations of this methodology are discussed in the discussion section.

### Validation

Our model was validated using data from the Alzheimer’s Disease Neuroimaging Initiative (ADNI). Correction for diet and exercise was excluded from the validation because these data were not available from the ADNI database. Only participants with a known APOE status and having sufficient data collected for analysis were included in the data analysis. The probability of AD symptoms was calculated using our tool from data supplied from AD participants (n = 250) and age-matched healthy controls (n = 1751). Mean “Risk of AD” at the current age was calculated for healthy controls and Mean “Risk of AD” at the time of AD onset was calculated for AD patients within the dataset.

## Results

**Fig 1:**
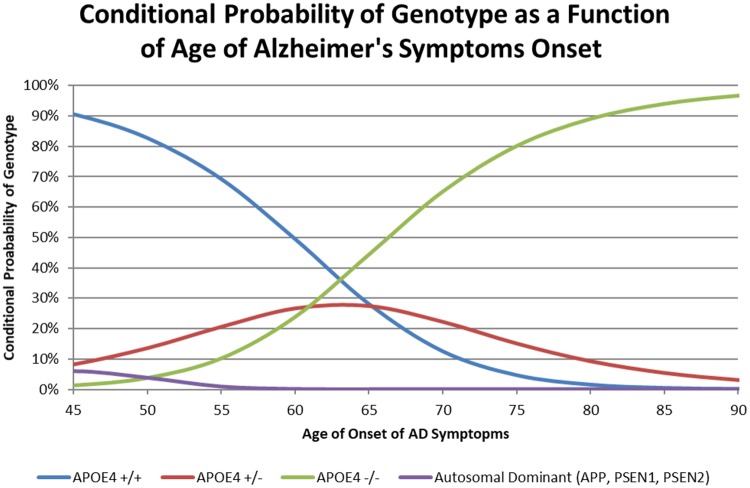
Theoretical Bayesian probability of APOE4 genotype based on age of AD onset. These functions form the basis for the P(AGE|Genotype) variable used in determining posterior probability P(Genotype|AGE). At early ages, the sum of the genetics add up to above 100% since the probability of autosomal dominant genes does not figure into the probabilities of have any of the APOE4 genotypes, which do add up to 100%. This graph is obtained using the conditional probability equation of ${P(Genotype|AGE)}_{g}=\frac{P({AGE|Genotype}_{g})\;∙{PopFreq}_{g}}{P(AGE)}$.

We begin our analysis by graphing the conditional probability function, P(AGE|Genotype), of a subject having a given APOE4 genotype based on their age of onset (Fig 1). The APOE4 +/+ genotype is associated with an earlier onset than +/- and -/- genotypes, however it occurs much less frequently in the population than those genotypes. An early AD onset is associated with +/+ genotype, however the probability that the subject has the APOE4 +/+ genotype decreases rapidly with reported age of onset given its low prevalence in the population. Additionally, early AD onset is associated with the autosomal dominant genes APP, PSEN1 and PSEN2. Given the genetic infrequency of these genes, the probability that a subject having an autosomal dominant genotype is still quite low, even when AD symptoms appear early. Conversely, the APOE4 -/- genotype is far more common in the population and is associated with later AD onset, if AD onset even occurs.

Table 2 contains a prediction of APOE4 genotype for a hypothetical individual to serve as an example.

**Table 2:** Example of hierarchical Bayesian model to estimate APOE4 genotype of a subject based on the age and age of AD onset of the subject and his parents and grandparents.

Paternal					Maternal				
**Grandfather**	82 87	**Grandmother**	-- 75	AD Onset Age	**Grandfather**	-- 79	**Grandmother**	67 77	AD Onset Age
+/+ 1%, +/- 8%, -/- 91%		+/+ 4%, +/- 9%, -/- 87%		APOE4	+/+ 4%, +/- 9%, -/- 87%		+/+ 21%, +/- 26%, -/- 53%		APOE4
**Father**			64 75	AD Onset Age	**Mother**			85 97	AD Onset Age
+/+ 3%, +/- 48%, -/- 49%				APOE4	+/+ 0%, +/- 25%, -/- 75%				APOE4
			**Subject**		-- 45	AD Onset Age			
			+/+ 3%, +/- 33%, -/- 64%			APOE4			

The calculated estimated APOE4 genotype is noted in the bottom of the cell for each individual and is calculated in accordance with the methodology in the Methods section. Onset is the age of AD onset. If there is no history of AD, then the field is blank. Age is the current age of the individual or the age of death, although this variable is not used in the analysis.

**Fig 2:**
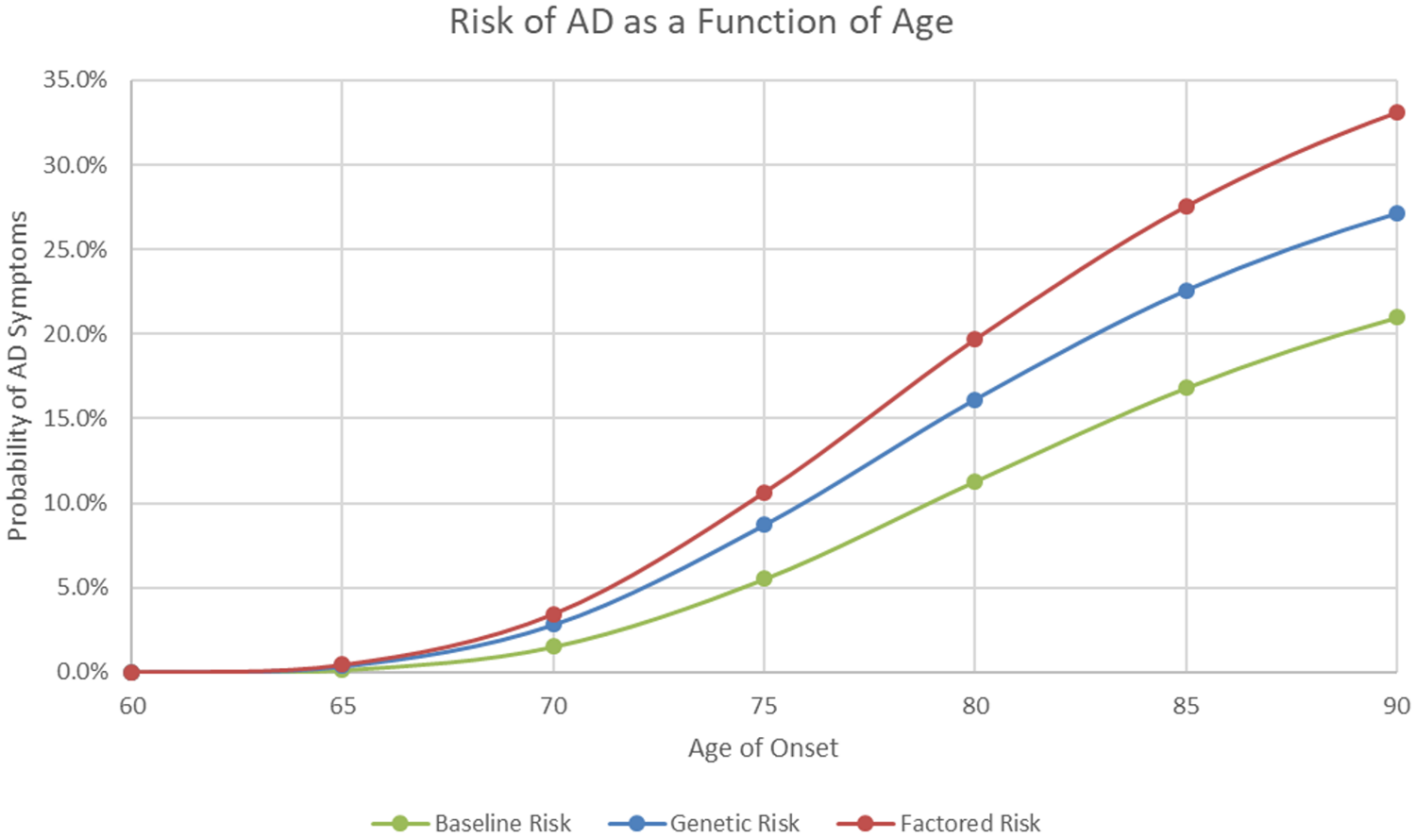
Gompertz AD onset risk function for the general population (“baseline”), the subject’s estimated APOE status (“genetic risk”), and risk factoring in known risk factors (“factored risk”). Notice the risk increases steadily until approximately age 80, at which point the risk grows at a slower rate. This is because there is a probability that this individual will never have AD. The baseline risk is calculated by applying the model to the “average” person, that has APOE4 genotype probability status equal to that of the population.

Once the APOE4 genotype is estimated, a “baseline” age of onset Gompertz function can be plotted based on the previously calculated parameters of A, k, and x weighted in accordance with the probability of the individual having each of the +/+, +/-, -/- genotypes (Fig 2).

Finally, additional risk factors were added to modify the amplitude of the Gompertz AD risk function. In our example, the individual in the example above is a white male with a history of diabetes and no traumatic brain injury with a high school education. He exercises regularly and eats a typical “American” diet. His hazard ratios are shown in Table 3.

**Table 3:** 

Factor	Hazard Ratio
European	1.0
History of T2DM	1.41
No History of TBI	1.0
Post-secondary Education	0.86
Regular Exercise	0.84
Non-Mediterranean diet	1.12
**TOTAL**	**1.22**

List of hazard ratios used for adjusting the Gompertz age of AD onset function for our example.

The Gompertz AD onset function is multiplied by a factor of 1.22 to obtain a factored AD onset risk function (Fig 2).

Table 4 contains a list of the most well-established factors that affect AD onset with their corresponding hazard ratios and reference to the appropriate literature.

**Table 4:** 

Phenotype	Prevalence in North America	Hazard Ratio	Adjusted Hazard Ratio	References
**Race**				[14]
White	63%	1.25	0.973	
Black	14%	1.73	1.346	
Hispanic	14%	1.29	1.00	
Asian	6%	1.0	0.78	
Aboriginal	1%	1.42	1.11	
**History of T2DM**				[19,20]
No T2DM	74%	1.0	0.855	
T2DM	26%	1.62	1.41	
**History of TBI** *				[7]
No/Mild TBI	>99%	1.0	1.0	
Moderate TBI	<1%	2.3	2.2	
Severe TBI	<1%	4.5	4.5	
**Education**				[21]
Secondary or greater	68%	1.0	0.855	
Primary or less	32%	1.78	1.522	
**Physical Exercise**				[10]
None	25.7%	1.0	1.33	
Some	35.8%	0.71	0.94	
Regular	38.5%	0.63	0.84	
**Diet**				[10]
Mediterranean	73.1%	0.6	1.12	
Other	26.9%	1.0	0.67	

List of established AD risk factors and their previously published hazard ratios.

* Mild TBI is defined as no skull fracture and altered level of consciousness (LOC) <30 min. Moderate TBI is altered LOC >30 min, but <24 hrs. Severe TBI is altered LOC >24 hrs.

Finally, we validated our theoretical model to actual data acquired from the Alzheimer’s Disease Neuroimaging Initiative (ADNI) dataset. AD patients (n = 250, μ = 74.0±7.0) were age matched to healthy controls (n = 1751, μ = 76.0±7.0). Data from these patients were inputted into the AD prediction calculator and the probability of AD at the participants’ age (for healthy controls) or the age of AD onset (for AD participants) was calculated. The mean theoretical probability of displaying AD symptoms for healthy controls was 12.2% whereas the mean theoretical probability of displaying AD symptoms for AD patients was 15.9%.

## Discussion

In this work, we provide a theoretical framework based on a hierarchical Bayesian model for estimating an individual’s APOE4 genotype and further to estimate the probability of AD onset as a function of age based on a number of known factors that affect AD onset. AD is a progressive disease and is heavily dependent on APOE4 genotype [2,16,22], however a number of modifiable lifestyle factors are also implicated in its onset and progression [10].

We can apply this approach in a hierarchical manner to estimate the genotype of an individual based on the age of AD onset of his or her parents. In this case, the parents’ posterior probability of a given genotype is used as the prior probability for the subject’s genotype estimation. This methodology can then be applied to subsequent generations. In our model we only go back two generations from the subject to estimate the subject’s genotype. This is because it is rare for subjects to be familiar with the cognitive status of their great-grandparents. Additionally, cognitive history beyond two generations has little impact on the genotype estimation of a subject. It is worth noting that even if the subject is not familiar with an AD status of a parent or grandparent, the model simply assigns an APOE4 genotype estimation equal to the prior probability.

The strength of our work is that it provides a low cost rough estimation of AD onset that is accessible to members of the general public and provides concerned individuals lifestyle modification factors that affect the risk of AD. Secondly, the Bayesian approach to determining APOE4 genotype is a novel method of determining a specific genotype and could be adapted to the prediction of other disease such as BRCA genotypes for individuals with breast cancer.

In addition to the strengths of our work that we have identified, our work has a number of important limitations that must be identified:

1. We added the hazard ratios from different factors (i.e. education, diet, etc.) to obtain a cumulative hazard ratio. In reality, different hazards act in a confounding, not an additive, fashion. However, we found no individuals in our dataset that had the both of the greatest risk factors for AD (T2DM and TBI). The result of this limitation is that in the highly improbable case where an individual has a high probability of APOE4 +/+ genotype, low educational attainment, and a history of T2DM and TBI, the predicted AD onset would be in excess of 100% at a certain age. Whether a subject with multiple co-morbidities and such a poor prognosis would actually live to this theoretically certain age of AD onset would be the subject of conjecture.
2. Because this model relies heavily on the APOE4 genotype prediction, this methodology only estimates AD onset, not the onset of dementia of different aetiologies such as vascular dementia.
3. AD has overlapping diagnostic criteria with other aetiologies of dementia and differentiating AD is somewhat problematic. For this reason, an individual may have been incorrectly diagnosed with AD thereby influencing the APOE4 genotype estimation of his progeny.
4. This model makes no prediction of EOAD onset.

The difference between AD onset risk for healthy individuals (12.2%) vs the risk of AD for people who displayed AD symptoms (15.9%) highlights the highly sporadic nature of LOAD. Despite these limitations, this work can still serve as a theoretical framework for future studies with sufficient resources to conduct large scale clinical trials to validate the hazards for different factors and provide a more accurate tool for the prediction of AD onset.

## Supporting information

S1 File The raw data used in this report is attached as supporting information provided as an Microsoft excel file. (XLSX)
